# TECHNOLOGY-BASED INTERVENTIONS AND ASSESSMENT FOR OLDER ADULTS WITH COGNITIVE IMPAIRMENT

**DOI:** 10.1093/geroni/igac059.268

**Published:** 2022-12-20

**Authors:** JuYoung Park, Lillian Hung

## Abstract

During the COVID-19 pandemic, older adults with cognitive impairment experienced social isolation, stress, and challenges to stay healthy at home or in a long-term care facility. Technology-based interventions and assessment can be valuable in managing dementia at home before a crisis situation occurs, which can lessen caregiver burden and stress and improve quality of life for older adults with cognitive impairment. In the symposium, specific technology-based interventions (telepresence robot, online chair yoga, exergames, virtual cycling, video-conferencing platforms) and assessment (IOM2 biofeedback device) were used for older adults with cognitive impairment. We cultivated a novel interdisciplinary approach to emerging clinical entities of technology-based intervention and assessment for older adults with cognitive impairment. In the symposium, we will present a variety of technology-based clinical interventions. Our first study explored the experiences of virtual family visits in four Canadian long-term care homes, using a telepresence robot. Online survey, interviews, focus groups, and observations were conducted to explore the experience. The second study assessed feasibility of a remotely supervised online chair yoga (CY) intervention for older adults with dementia in Florida and explored the preliminary effects of CY on psychosocial outcomes in this population. The third study evaluated the ease of use and quality of cardiac data using IOM2 biofeedback device for older adults with dementia. Cardiac rhythms were analyzed from pulse data measured using the IOM2 biofeedback device (UNYTE). The fourth study was a scoping review to analyze evidence about online group-based exercise programs.

